# Age Distribution of Cases of 2009 (H1N1) Pandemic Influenza in Comparison with Seasonal Influenza

**DOI:** 10.1371/journal.pone.0021690

**Published:** 2011-07-01

**Authors:** Drosos E. Karageorgopoulos, Evridiki K. Vouloumanou, Ioanna P. Korbila, Anastasios Kapaskelis, Matthew E. Falagas

**Affiliations:** 1 Alfa Institute of Biomedical Sciences (AIBS), Athens, Greece; 2 Department of Medicine, Henry Dunant Hospital, Athens, Greece; 3 Department of Medicine, Tufts University School of Medicine, Boston, Massachusetts, United States of America; National Institutes of Health, United States of America

## Abstract

**Introduction:**

Several aspects of the epidemiology of 2009 (H1N1) pandemic influenza have not been accurately determined. We sought to study whether the age distribution of cases differs in comparison with seasonal influenza.

**Methods:**

We searched for official, publicly available data through the internet from different countries worldwide on the age distribution of cases of influenza during the 2009 (H1N1) pandemic influenza period and most recent seasonal influenza periods. Data had to be recorded through the same surveillance system for both compared periods.

**Results:**

For 2009 pandemic influenza versus recent influenza seasons, in USA, visits for influenza-like illness to sentinel providers were more likely to involve the age groups of 5–24, 25–64 and 0–4 years compared with the reference group of >64 years [odds ratio (OR) (95% confidence interval (CI)): 2.43 (2.39–2.47), 1.66 (1.64–1.69), and 1.51 (1.48–1.54), respectively]. Pediatric deaths were less likely in the age groups of 2–4 and <2 years than the reference group of 5–17 years [OR (95% CI): 0.46 (0.25–0.85) and 0.49 (0.30–0.81), respectively]. In Australia, notifications for laboratory-confirmed influenza were more likely in the age groups of 10–19, 5–9, 20–44, 45–64 and 0–4 years than the reference group of >65 years [OR (95% CI): 7.19 (6.67–7.75), 5.33 (4.90–5.79), 5.04 (4.70–5.41), 3.12 (2.89–3.36) and 1.89 (1.75–2.05), respectively]. In New Zealand, consultations for influenza-like illness by sentinel providers were more likely in the age groups of <1, 1–4, 35–49, 5–19, 20–34 and 50–64 years than the reference group of >65 years [OR (95% CI): 2.38 (1.74–3.26), 1.99 (1.62–2.45), 1.57 (1.30–1.89), 1.57 (1.30–1.88), 1.40 (1.17–1.69) and 1.39 (1.14–1.70), respectively].

**Conclusions:**

The greatest increase in influenza cases during 2009 (H1N1) pandemic influenza period, in comparison with most recent seasonal influenza periods, was seen for school-aged children, adolescents, and younger adults.

## Introduction

Since the spring of 2009, the globe has witnessed an influenza pandemic due to a variant H1N1 influenza virus [1]. The initial public health guidance and response was inevitably based on the evidence obtained from previous influenza pandemics. Subsequently, a wealth of information collected from various sources worldwide regarding the main epidemiological characteristics, clinical manifestations, and outcome of the new influenza pandemic has rapidly accumulated [2]. However, several important aspects of the true impact of 2009 (H1N1) pandemic influenza have not been accurately determined to guide appropriate public health responses [3].

In this regard, we sought to explore the differences between the 2009 (H1N1) pandemic influenza period and recent seasonal influenza periods regarding the age distribution of influenza cases.

## Methods

We searched, up to March 2010, in the official websites of major health organizations or institutions worldwide for publicly available surveillance data on the age distribution of influenza cases. Specifically, we searched in the websites of the World Health Organization, the European Centre for Disease Prevention and Control, the United Kingdom Health Protection Agency and Office of National Statistics, the French Institute for Public Health Surveillance, the Swedish Institute for Communicable Disease Control, the United States Centers for Disease Control and Prevention (CDC) and the Department of Defense Global Emerging Infections Surveillance and Response System, the Public Health Agency of Canada, the Australian Government Department of Health and Ageing and the National Notifiable Diseases Surveillance System, the New Zealand Ministry of Health and the Institute of Environmental Science and Research, the South African National Institute for Communicable Diseases, the Pan American Health Organization, the Argentinean Ministry of Health, the Mexican Department of Health, the Chilean Ministry of Health, and the Brazilian Ministry of Health. We also searched for additional relevant influenza surveillance data, through web links from the above websites. Finally, we retrieved official data on the type and antigenic characterization of circulating influenza viruses during each influenza season and country analyzed.

We included in our analysis epidemiological data referring to the 2009 (H1N1) pandemic influenza period (from mid April 2009 until the end of 2009) and one or more of the most recent influenza seasons, provided that they were recorded with the same surveillance system and similar methodology. We included data on influenza cases that had been diagnosed either clinically or with appropriate relevant laboratory methods, regardless of the level of health-care they received. We synthesized the retrieved data into meaningful and comparable age groups. We calculated the percentage of cases in the different age groups for both the pandemic and the seasonal influenza periods. We constructed graphical representations of the percentile distribution of cases into age groups between the pandemic and, cumulatively, the seasonal influenza periods. We also calculated the percentage difference in the recorded number or rate of cases, between the pandemic and the seasonal influenza periods, for each age group.

### Statistical analysis

We compared the distribution of cases into age groups between the pandemic and, cumulatively, the seasonal influenza periods, using the chi-square test. A p value <0.05 was considered statistically significant. We also calculated the odds ratio (OR) and 95% confidence interval (CI) for a case of influenza to belong to each specific age group compared with a reference age group, between the pandemic and, cumulatively, the seasonal influenza periods. We used the older age group in each dataset as the reference group. For the above analyses, we used the SPSS Statistics v.17.0 software (SPSS Inc., Chicago, IL).

## Results

**Figure 1:**
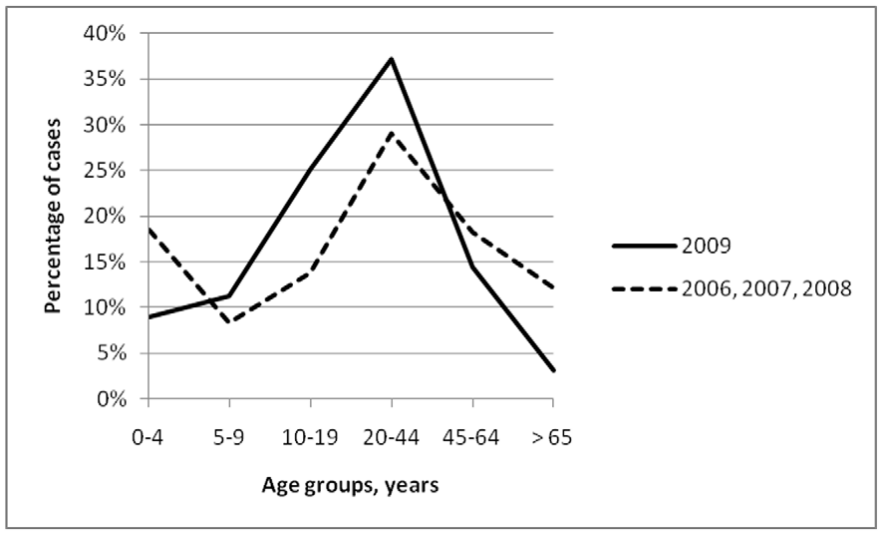
Percentile distribution by age group of notifications for laboratory-confirmed influenza in Australia, during 2009 (H1N1) pandemic influenza and the 3 previous influenza seasons combined.

**Figure 2:**
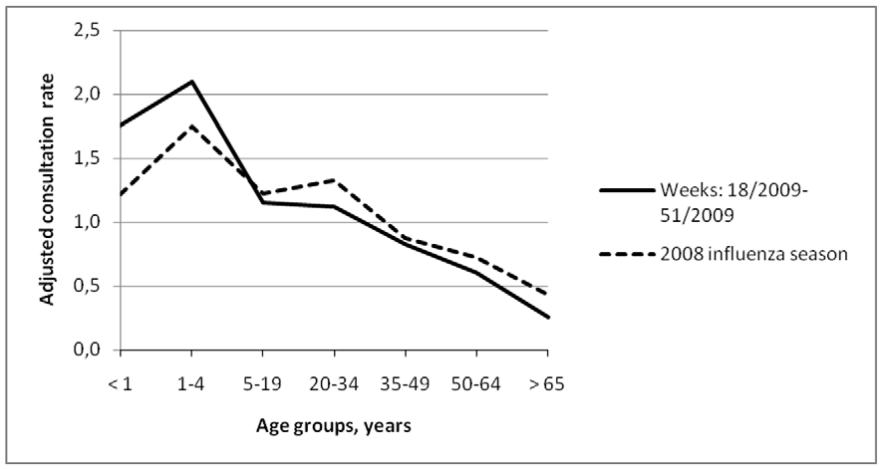
Adjusted sentinel average weekly consultation rate for influenza-like illness by age group in New Zealand, during 2009 (H1N1) pandemic influenza and the previous influenza season. Consultation rate by age group is shown as times X consultation rate for total population.

**Figure 3:**
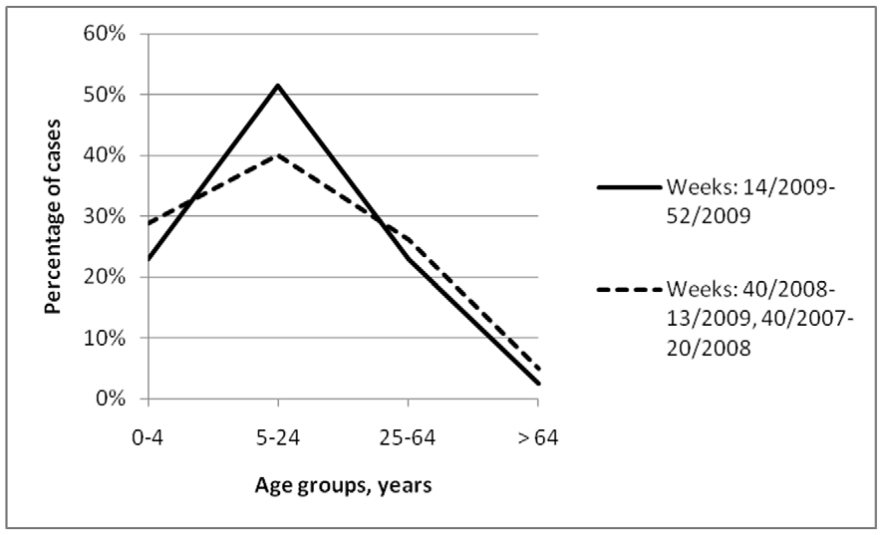
Percentile distribution by age group of visits for influenza-like-illness to sentinel providers, in the United States of America, during 2009 (H1N1) pandemic influenza and the 2 previous influenza seasons combined.

**Figure 4:**
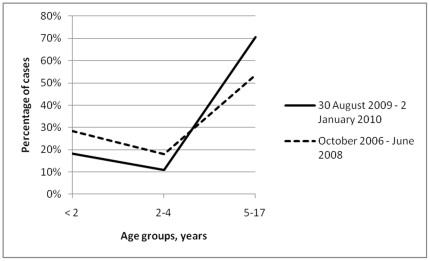
Percentile distribution by age group of pediatric deaths associated with laboratory-confirmed influenza, in the United States of America, during 2009 (H1N1) pandemic influenza and 2 previous influenza seasons combined.

We identified comparable data on the age distribution of influenza cases between the 2009 (H1N1) pandemic period and most recent seasonal influenza periods that were provided by the CDC, the Australian Government Department of Health and Ageing, and the New Zealand Institute of Environmental Science and Research. In Tables 1, 2, 3, 4, 5, we present the actual data that we retrieved and combined into age groups. We also present official data on the prevalence of different viral types and subtypes that circulated during each of the seasonal influenza periods and country evaluated. Additionally, we present in Tables 1, 2, 3, 4, 5 and in Figures 1, 2, 3, 4, the percentile distribution of cases into different age groups for both the pandemic and the compared seasonal influenza periods. Moreover, we present the percentage difference between the absolute number of the recorded influenza cases (or rate per unit of population) between the pandemic influenza period and the average for the seasonal influenza periods. Finally, the odds ratio for a case of influenza to belong to each specific age group compared with the reference group in the pandemic versus the seasonal influenza periods is also shown in Tables 1, 2, 3, 4, 5. More specific data are presented below, according to the type of influenza diagnosis and severity of cases.

**Table 1:** Notifications of laboratory-confirmed influenza for different age groups, during the 2009 (H1N1) pandemic influenza period and recent seasonal influenza periods, in Australia.*

Period	Viral types and subtypes	Age groups, n (%)						
		*0–4 y*	*5–9 y*	*10–19 y*	*20–44 y*	*45–64 y*	*>65 y*	*Unknown*
2009	2009 (H1N1) pandemic influenza	4014 (8.9%)	5060 (11.2%)	11318 (25.1%)	16720 (37.1%)	6517 (14.5%)	1393 (3.1%)	10 (<0.1%)
2008	Influenza A: 43% (H1N1: 27%, H3N2: 73%), Influenza B: 54%	1335 (14.6%)	730 (8.0%)	1428 (15.6%)	2748 (30.1%)	1785 (19.5%)	1109 (12.1%)	4 (0.04%)
2007	Influenza A: 93% (H1N1: 37%, H3N2: 63%), Influenza B: 7%	2241 (21.5%)	936 (9.0%)	1253 (12.0%)	3051 (29.2%)	1770 (16.9%)	1195 (11.4%)	1 (0.01%)
2006	Influenza A: 65% (H1N1: 6%, H3N2: 94%), Influenza B: 35%	653 (20.1%)	230 (7.1%)	461 (14.2%)	817 (25.1%)	618 (19.0%)	476 (14.6%)	0
Seasonal influenza periods combined		4229 (18.5%)	1896 (8.3%)	3142 (13.8%)	6616 (29.0%)	4173 (18.3%)	2780 (12.2%)	5 (<0.1%)
		**Percentage difference in notifications between 2009 and average of 2008, 2007 & 2006 seasons**						
		185%	701%	981%	658%	369%	50%	
**Comparison of the age distribution between pandemic influenza and the seasonal influenza periods combined**		**Odds ratio (95% confidence interval) for a case of influenza to belong to a specific age group, in the pandemic versus seasonal influenza periods**						
*P*<0.001		1.89 (1.75–2.05)	5.33 (4.90–5.79)	7.19 (6.67–7.75)	5.04 (4.70–5.41)	3.12 (2.89–3.36)	Reference group	

Abbreviations: y: years.

* Data are from Australian National Notifiable Diseases Surveillance System (http://www9.health.gov.au/cda/Source/CDA-index.cfm).

**Table 2:** Sentinel average weekly consultation rate for influenza-like illness per 100000 patient population (calculated absolute number of annual sentinel consultations^#^) for different age groups, during the 2009 (H1N1) pandemic influenza period and recent seasonal influenza periods, in New Zealand.*

Period	Viral types and subtypes	Age groups						
		*<1 y*	*1–4 y*	*5–19 y*	*20–34 y*	*35–49 y*	*50–64 y*	*>65 y*
Wks 18/2009-53/2009	2009 (H1N1) pandemic influenza	136.5 (278)	163.1 (1283)	89.9 (2890)	87.4 (2486)	64.3 (2104)	47.2 (1132)	20.1 (359)
Wks 18/2008-40/2008	Influenza A: 42% (H1N1: 2%, H3N2: 98%), Influenza B: 58%	63.6 (69)	91.5 (380)	64.2 (1090)	69.7 (1047)	45.8 (792)	38.0 (481)	22.5 (212)
		**Percentage difference in consultation rate between 2009 and 2008**						
		115%	78%	40%	25%	40%	24%	–11%
**Comparison of the age distribution between pandemic influenza and the seasonal influenza periods combined**		**Odds ratio (95% confidence interval) for a case of influenza to belong to a specific age group, in the pandemic versus seasonal influenza periods**						
*P*<0.001		2.38 (1.74–3.26)	1.99 (1.62–2.45)	1.57 (1.30–1.88)	1.40 (1.17–1.69)	1.57 (1.30–1.89)	1.39 (1.14–1.70)	Reference group

Abbreviations: y: years, wks: weeks.

* Data are from New Zealand Institute of Environmental Science and Research (http://www.surv.esr.cri.nz/index.php).

# Calculated data should be considered as approximate.

**Table 3:** Visits for influenza-like-illness reported by sentinel providers for different age groups, during the 2009 (H1N1) pandemic influenza period and recent seasonal influenza periods, in the United States of America.*

Period	Viral types and subtypes	Age groups, n (%)			
		*0–4 y*	*5–24 y*	*25–64 y*	*>64 y*
Wks 14/2009 – 52/2009	2009 (H1N1) pandemic influenza	212324 (23.0%)	475038 (51.4%)	212340 (23.0%)	23954 (2.6%)
Wks 40/2008 – 13/2009	Influenza A: 67.3% (H1N1: 89.8%, H3N2: 10.2%), Influenza B: 32.7%	83992 (29.9%)	117573 (41.8%)	67044 (23.8%)	12212 (4.3%)
Wks 40/2007 – 20/2008	Influenza A: 71% (H1: 26%, H3: 74%), Influenza B: 29%	106581 (28.0%)	147222 (38.7%)	105913 (27.8%)	20265 (5.3%)
Seasonal influenza periods combined		190573 (28.8%)	264795 (40.1%)	172957 (26.2%)	32477 (4.9%)
		**Percentage difference in the number of visits between 2009 and the average of 2008–2009 & 2007–2008 seasons**			
		123%	259%	146%	48%
**Comparison of the age distribution between pandemic influenza and the seasonal influenza periods combined**		**Odds ratio (95% confidence interval) for a case of influenza to belong to a specific age group, in the pandemic versus seasonal influenza periods**			
P<0.001		1.51 (1.48–1.54)	2.43 (2.39–2.47)	1.66 (1.64–1.69)	Reference group

Abbreviations: y: years, wks: weeks.

* Data are from US Centers for Disease Control and Prevention; US Outpatient Influenza-like Illness Surveillance Network (ILINet) (http://www.cdc.gov/flu/weekly/fluactivity.htm).

**Table 4:** Laboratory-confirmed influenza-associated hospitalization rate for different age groups, during the 2009 (H1N1) pandemic influenza period and recent seasonal influenza periods, in the United States of America.*

Period	Viral types and subtypes	Age groups, rate per 10000 general population					
		*0–23 m*	*2–4 y*	*5–17 y*	*18–49 y*	*50–64 y*	*≥65 y*
1 Sep 2009 – 2 Jan 2010∧	2009 (H1N1) pandemic influenza	5.7		2.4	2.3	2.9	2.2
15 Apr 09 – 29 Aug 09	2009 (H1N1) pandemic influenza	2.4	0.9	0.8	0.5	0.6	0.5
1 Oct 2008 –28 Mar 2009	A: 67.3% (H1N1: 89.8%, H3N2: 10.2%), Influenza B: 32.7%	2.8		0.5	0.3	0.4	1.0
30 Sep 2007 – 3 May 2008	Influenza A: 71% (H1: 26%, H3: 74%), Influenza B: 29%	4.0		0.6	ΝΑ	NA	NA
		**Percentage difference in hospitalization rate between Sep 2009 –Jan 2010 and Oct 2008 – Mar 2009**					
		104%		380%	667%	625%	120%

Abbreviations: m: months, NA: non-available, y: years, wks: weeks.

* Data are from US Centers for Disease Control and Prevention; Emerging Infections Program (EIP) (http://www.cdc.gov/flu/weekly/fluactivity.htm).

∧ Rates for new EIP sites are not included.

**Table 5:** Laboratory-confirmed influenza-associated pediatric deaths in different age groups, during the 2009 (H1N1) pandemic influenza period and recent seasonal influenza periods, in the United States of America.*

Period	Viral types and subtypes	Age groups, n (%)		
		*<2 y*	*2–4 y*	*5–17 y*
30 Aug 2009 – 2 Jan 2010	2009 (H1N1) pandemic influenza	42 (18.3%)	25 (10.9%)	162 (70.7%)
Oct 2007 – Jun 2008	Influenza A: 71% (H1: 26%, H3: 74%), Influenza B: 29%	23 (27.7%)	18 (21.7%)	42 (50.6%)
Oct 2006 – May 2007	Influenza A: 79% (H1: 62%, H3: 38%), Influenza B: 21%	20 (29.4%)	9 (13.2%)	39 (57.4%)
Seasonal influenza periods combined		43 (28.5%)	27 (17.9%)	81 (53.6%)
		**Percentage difference in pediatric deaths between Aug 2009 –Jan 2010 and the average of 2007–2008 & 2006–2007 seasons**		
		95%	85%	300%
**Comparison of the age distribution between pandemic influenza and the seasonal influenza periods combined**		**Odds ratio (95% confidence interval) for a case of influenza to belong to a specific age group, in the pandemic versus seasonal influenza periods**		
*P* = 0.003		0.49 (0.30–0.81)	0.46 (0.25–0.85)	Reference group

Abbreviations: y: years.

Data are from US Centers for Disease Control and Prevention; Influenza-Associated Pediatric Mortality Surveillance System (http://www.cdc.gov/flu/weekly/fluactivity.htm).

### Unselected laboratory-confirmed cases of influenza

In Australia, the National Notifiable Diseases Surveillance System provided data regarding the number of cases of laboratory-confirmed influenza (Table 1). In 2009 (pandemic influenza period) the cases of laboratory-confirmed influenza (total of 45032) increased by 491% compared with the average number of those recorded during the 3 preceding seasonal influenza periods (2006–2008; average of 7614 per year). The distribution of influenza cases into age groups differed between the pandemic influenza period and the seasonal influenza periods combined (*P*<0.001; Figure 1). For the pandemic influenza period versus the seasonal influenza periods, a case of influenza was more likely to belong into the age group of 10–19 years (OR: 7.19, 95% CI: 6.67–7.75), 5–9 years (OR: 5.33, 95% CI: 4.90–5.79), 20–44 years (OR: 5.04, 95% CI: 4.70–5.41), 45–64 years (OR: 3.12, 95% CI: 2.89–3.36), and 0–4 years (OR: 1.89, 95% CI: 1.75–2.05) compared with the reference age group of those more than 65 years.

### Cases of influenza-like illness

In New Zealand, the Institute of Environmental Science and Research, a governmental research institute, provided data regarding the average weekly consultation rate for influenza-like illness in sentinel practices per 100000 patient population reported by these practices (Table 2). In 2009 (pandemic influenza period) the average weekly sentinel consultation rate for influenza-like illness (77.9 per 100000 patient population) increased by 48.7% compared with 2008 (seasonal influenza period; 52.4 per 100000 patient population). Figure 2 presents the average weekly consultation rate for influenza-like illness in sentinel practices by age group, adjusted to the respective consultation rate for the total population, for 2009 in comparison with 2008.

We used the above presented data on the average weekly sentinel consultation rate for influenza-like illness in New Zealand to approximately calculate the absolute number of cases of influenza-like illness reported by the sentinel practices during the pandemic and seasonal influenza periods that we evaluated (Table 2). Specifically, we multiplied the age-specific weekly consultation rate by the total number of weeks that influenza surveillance was in effect for each influenza period (36 weeks for 2009 and 23 weeks for 2008) and the total age-specific patient population examined in the sentinel practices. According to the official reports by the New Zealand Institute of Environmental Science and Research, the total age-specific patient population examined in the sentinel practices was not recorded, but derived from the total patient-population examined in these practices (402884 for 2009 and 333150 for 2008), assuming that it had the same age distribution as the New Zealand general population, according to the census data. We used relevant data from the 2006 New Zealand census in this regard.

The distribution of influenza-like illness cases into age groups differed between the 2009 pandemic influenza period and the 2008 seasonal influenza period (*P*<0.001). For the pandemic influenza period versus the seasonal influenza period, a case of influenza was more likely to belong into the age group of less than 1 year (OR: 2.38, 95% CI: 1.74–3.26), 1–4 years (OR: 1.99, 95% CI: 1.62–2.45), 35–49 years (OR: 1.57, 95% CI: 1.30–1.89), 5–19 years (OR: 1.57, 95% CI: 1.30–1.88), 20–34 years (OR: 1.40, 95% CI: 1.17–1.69), and 50–64 years (OR: 1.39, 95% CI: 1.14–1.70), compared with the reference group of those more than 65 years.

In the United States of America (USA), the CDC provided data regarding the number of visits to sentinel providers for influenza-like illness (Table 3). During the 2009 (H1N1) pandemic influenza period visits to sentinel providers for influenza-like illness (n = 923656) increased by 180% compared with the average of those recorded cumulatively over the 2 preceding seasonal influenza periods (2008–09 and 2007–08, respectively, average of 330401 visits per year). The distribution of visits for influenza-like illness to sentinel providers differed between the 2009 pandemic influenza period and the 2008 seasonal influenza period (*P*<0.001; Figure 3). For 2009 versus the 2007 and 2008 influenza seasons combined, a case of influenza-like illness recorded by sentinel providers was more likely to belong in the age group of 5–24 years (OR: 2.43, 95% CI: 2.39–2.47), 25–64 years (OR: 1.66, 95% CI: 1.64–1.69), and 0–4 years (OR: 1.51, OR: 1.48–1.54) compared with the reference age group of those more than 64 years (OR: 0.515, 95% CI: 0.506–0.524).

### Cases hospitalized with influenza

In the USA, the CDC provided data regarding the laboratory-confirmed influenza-associated hospitalization rate for the general population (Table 4). Relevant data for the 2009 (H1N1) pandemic influenza period were separately reported for the period between 15 April – 29 August 2009 and the period between 1 Sep 2009 – 2 Jan 2010. This was related to a change in the relevant surveillance system with addition of new sites. For the latter period, we included surveillance data provided only by the old sites, so that they could be directly comparable with prior relevant surveillance data. The age distribution of cumulative laboratory-confirmed influenza-associated hospitalization rates did not appear to substantially differ between the two 2009 (H1N1) pandemic influenza periods mentioned above. The highest relevant rate for both these periods was observed in the age group of 0–4 years, whereas rates in the remaining age groups were approximately 30–50% lower.

We compared the age distribution of laboratory-confirmed influenza-associated hospitalization rates between the pandemic period of 1 Sep 2009 – 2 Jan 2010 and the whole 2008–2009 influenza season, because corresponding age-specific data were provided only for these periods. During the above-mentioned 2009 (H1N1) pandemic influenza period, compared with the 2008–2009 seasonal influenza period, the cumulative laboratory-confirmed influenza-associated hospitalization rate increased primarily for the age groups of 18–49 years and 50–64 years (increase by 667% and 625% in the hospitalization rate, respectively); the increase in the hospitalization rate was relatively lower for the age group of 5–17 years (380%), followed by the groups of those more than 65 years and those up to 4 years of age (120% and 104%, respectively). As we could not reliably retrieve accurate data regarding the size and age distribution of the reference population for the hospitalization rates analyzed above, we did not proceed to calculate the respective raw hospitalization data.

### Deaths associated with influenza

In USA, the CDC provided data regarding the number of laboratory-confirmed influenza-associated pediatric deaths (Table 5). For the 2009 (H1N1) pandemic influenza period relevant age-specific cumulative data were provided only for the period of 30 August 2009 – 2 January 2010. During these last 4 months of 2009 (pandemic influenza period) laboratory-confirmed influenza-associated pediatric deaths (n = 229) increased by 203% compared with the yearly average of the whole 2007–2008 and 2008–2009 seasonal influenza periods (average of 75.5 deaths per year). The distribution of pediatric deaths into age groups differed significantly between the pandemic influenza period and the seasonal influenza periods compared (*P* = 0.003, Figure 4). For the pandemic influenza period versus the seasonal influenza periods combined, pediatric deaths associated with laboratory-confirmed influenza were less likely to be noted in the age groups of 2–4 years (OR: 0.46, 95% CI: 0.25–0.85) and of those less than 2 years (OR: 0.49, 95% CI: 0.30–0.81), compared with the reference group of 5–17 years.

## Discussion

A common finding in the surveillance data from major national health organizations worldwide that we identified for the purposes of this study is that the age group of elderly individuals (more than 65 years of age) was the least one affected during the 2009 (H1N1) pandemic influenza period, in comparison with the most recent seasonal influenza periods. This particularly refers to laboratory-confirmed disease incidence and presentation with influenza-like illness to sentinel providers, according to data recorded in different countries. Regarding the age groups mostly affected by 2009 (H1N1) pandemic influenza in comparison with the most recent seasonal influenza periods, we noted certain differences according to the level of influenza diagnosis (clinical or laboratory-confirmed), the severity of cases (outpatients, hospitalized, or deceased), and country. For 2009 (H1N1) pandemic influenza, in comparison with seasonal influenza, laboratory-confirmed influenza cases and laboratory-confirmed influenza-associated hospitalizations mostly increased for school-age children, adolescents, and younger adults, according to data from Australia and USA, respectively. Pediatric deaths associated with laboratory-confirmed influenza increased mostly for school-age children and adolescents, according to US data. The greatest increase in influenza-like illness presentations to sentinel providers was noted for school-age children, adolescents and young adults in USA, while, in New Zealand, this was noted for infants and preschool children.

Differences in the age distribution of cases of 2009 (H1N1) pandemic influenza in comparison with seasonal influenza can be attributed to differences in the degree of preexisting specific immunity in different age groups. Specifically, elderly individuals appear to have had protective immunity more frequently than other age groups against the 2009 (H1N1) pandemic influenza virus due to prior immunologic encounters with antigenically similar viruses many years before. Increasing age has been positively associated with the presence of cross-reactive antibodies against the 2009 (H1N1) pandemic influenza virus, which can prevent infection [4]. Still, specific immunity from memory T-cells could also have been present in a substantial percentage of the general population due to shared relevant antigenic epitopes between 2009 (H1N1) pandemic influenza virus and recent seasonal influenza A(H1N1) viruses and vaccine strains [5], [6]. However, cellular immune responses can be relatively weak in the elderly compared with younger individuals, and this could be associated with higher severity of influenza once infection occurs [7], [8].

Although 2009 (H1N1) pandemic influenza, compared with seasonal influenza, appears to have affected mainly school-age children, adolescents, and younger adults, regarding outpatient presentations for influenza-like illness in USA or incidence of laboratory-confirmed disease in Australia, there appears to have been a shift towards young and middle-aged adults for hospitalizations in USA. This could be explained by the more frequent co-existence of known risk factors for influenza in the latter age groups, compared with younger patients [9]. Conditions like pregnancy and obesity that have been associated with more serious 2009 (H1N1) pandemic influenza requiring hospitalization are also found more frequently among young and middle-aged adults [10], [11].

Similar observations as the ones made in our analysis regarding a greater impact of the 2009 (H1N1) pandemic influenza on younger patients, compared with seasonal influenza, have also been made for other influenza pandemics [12], [13]. A recent study that comparatively evaluated the 2009 (H1N1) influenza pandemic with select past seasonal H1N1 and H3N2 influenza A epidemics in USA and France did not identify statistically significant differences in the age distribution of the recorded influenza-like illness cases [14]. This study found, however, that, for the 2009 (H1N1) pandemic period, influenza-associated mortality was higher for those younger than 60 years, particularly for those younger than 20 years. The authors of this study suggested that, both in seasonal influenza epidemics as well as in influenza pandemics school-age, children have the highest contact rates, and that during the 2009 (H1N1) influenza pandemic mortality shifted towards those younger than 60 years of age due to lack of preexisting protective immunity.

Similar findings regarding the impact of 2009 (H1N1) influenza pandemic by age in terms of mortality were noted in another study that modeled excess pneumonia & influenza mortality in the general population in the USA during 2009–2010 and the past 47 influenza seasons [15]. For the age groups of <15 years and 15–24 years, excess pneumonia & influenza mortality during the fall wave of the (H1N1) 2009 influenza pandemic exceeded that of all previous influenza seasons, including the 1968–1969 influenza pandemic. For the age group of 25–64 years excess pneumonia and influenza mortality during the fall wave of the 2009 (H1N1) influenza pandemic was second only to the 1968–1969 influenza pandemic, while for the age group of those >65 years excess mortality was in the range of what has been observed for seasonal influenza periods that H1N1 viruses predominate. An additional study has noted that the mean age of influenza-related deaths in the USA during the 2009 (H1N1) pandemic (37.4 years) was considerably lower than the average for typical A/H3N2 influenza seasons (75.7 years), as well as the 1968 and 1957 pandemics (62.2–64.6 years), while it relatively close to the 1918 pandemic (27.2 years) [16].

Seroprevalence studies evaluating the temporal changes in the prevalence of antibodies against the 2009 (H1N1) pandemic influenza virus in the community have helped in the clarification of the evolution of the disease amongst different age groups. A study that applied statistical modeling to evaluate sequential seroprevalence data from England noted that during the second wave of 2009 (H1N1) pandemic influenza (September 2009 to February 2010) the cumulative incidence of infection was higher in the age group of 5–14 years, followed by the age group of 1–4 years, and those of 15–24 and 25–44 years [17]. Similar findings were noted in another study that evaluated differences in the seroprevalence of antibodies against 2009 (H1N1) pandemic influenza virus before and after the first wave of 2009 (H1N1) pandemic influenza in different age groups in England [18]. Another study from New Zealand that evaluated differences in seroprevalence data for antibodies against 2009 (H1N1) pandemic influenza virus before and after the 2009 (H1N1) pandemic influenza period noted that age was independently associated with the risk of infection. Individuals in the age groups of 5–19 and 1–4 years were significantly more likely to have developed high titers of specific antibodies compared with the reference group of 40–59 years [19]. The age distribution of influenza cases is expected to shift to older age groups as the pandemic progresses because of attainment of high rates of natural immunity in the younger age groups that were hit first [20]. It should be taken into consideration, however, that many of the seropositive cases in the above seroprevalence studies refer to asymptomatic or minimally symptomatic cases.

The findings of our analysis should be interpreted with caution for several reasons. First, the recorded cases of influenza reflect only a small subset of the actual clinical symptomatic cases, the majority of which is not expected to seek medical care [21]. Cases requiring admission in the intensive care unit or with a fatal outcome were also not adequately assessed, because we did not identify appropriate relevant data.

The comparative data we identified on the age distribution of cases of 2009 (H1N1) pandemic influenza and seasonal influenza, recorded by the same surveillance systems, derived from a relatively small number of sources. The data we included referred to developed countries (USA, Australia, and New Zealand), which have similar socioeconomic conditions, health care systems, and age distribution of the total population. They cannot be highly representative of countries under development, in many of which the life expectancy of the population is lower and the birth rate is greater, and, thus, the percentage of younger individuals in the population is higher. Differences between countries could also exist regarding the epidemiology of influenza, the surveillance and diagnostic methods used, including the definitions for syndromic surveillance, and the attitude of the public, healthcare professionals and administrative organizations towards influenza [2]. The data we analyzed refer to countries of both the northern and the southern hemisphere, in which the first wave of the 2009 (H1N1) pandemic influenza began in the summer and winter season, respectively.

Apart from biological differences in the disease characteristics [22], several factors could account for differences in influenza surveillance data in the pandemic compared with the seasonal influenza periods analyzed. These potentially include greater alertness of surveillance organizations, use of better diagnostic methods (such as real-time RT PCR instead of antigen-based tests), increased public awareness of influenza leading to enhanced health-seeking behavior, greater sensitivity of health care professionals in pursuing diagnosis and treatment of influenza, and different susceptibility to antiviral drugs.

An important relevant consideration refers to potential differences in vaccination coverage of different age groups of the general population between the pandemic and seasonal influenza periods. This is plausible given the differences in relevant public health recommendations and vaccination policies. Still, for most of the 2009 (H1N1) pandemic influenza period examined in the context of this study, the specific influenza vaccine was not available, and the vaccination coverage of the general population was considerably low in most countries [23]. The degree in which the above-mentioned parameters could have differentially affected different age groups in the pandemic compared with seasonal influenza periods, and thus confound our comparative analysis, is difficult to estimate.

An additional parameter, however, that can affect the age distribution of seasonal influenza cases is the predominant influenza strain in a certain region. H1 influenza A strains tend to have greater predilection for younger individuals compared with H3 strains, while influenza B is typically associated with milder illness than influenza A [24]. We summed data from more than one seasonal influenza periods, where available, so that the influence of this parameter could be mitigated. The differences in the predominant influenza types and subtypes during the comparator seasonal influenza periods could explain the differences in the comparative impact of 2009 (H1N1) pandemic influenza by age group between USA and New Zealand regarding visits for influenza-like illness to sentinel providers observed in our study (Tables 2–3). Finally, we should mention that we did not adjust the actual number of influenza cases by age group to the total population in each specific age group, because the compared periods differed only by 1–3 years, during which the age distribution of the population is not expected to have considerably changed.

In conclusion, the comparative analysis of publicly available data from 3 different countries, recorded by the same surveillance systems, shows that during 2009 (H1N1) influenza pandemic period the age distribution of cases shifted towards children, adolescents, and younger adults, in comparison with recent seasonal influenza periods. The comparative burden of the pandemic influenza might have been greater for young and middle-aged adults when hospitalizations are concerned. These data could aid public health authorities to better organize appropriate response strategies for future pandemics.
